# Immunophenotype in orofacial granulomatosis with and without Crohn’s disease

**DOI:** 10.4317/medoral.20187

**Published:** 2014-09-30

**Authors:** Gita Gale, Sofia Östman, Robert Saalman, Esbjörn Telemo, Mats Jontell, Bengt Hasséus

**Affiliations:** 1 Department of Oral Medicine and Pathology, Institute of Odontology; 2 Department of Infectious Diseases, Institute of Biomedicine; 3 Department of Paediatrics, Institute of Clinical Sciences; 4 Department of Rheumatology and Inflammation Research, Institute of Medicine, The Sahlgrenska Academy, University of Gothenburg, Gothenburg, Sweden

## Abstract

Objectives: The aim of this investigation was to characterise and compare the inflammatory infiltrates in patients with orofacial granulomatosis solely (OFG-S) and OFG with coexisting Crohn’s disease (OFG+CD). Study Design: Biopsy specimens with granulomas were obtained from patients with OFG-S (n=11) and OFG+CD (n=11) and immunostained with antibodies against CD1a, CD3, CD4, CD8, CD11c, CD20, CD68 and mast cell tryptase, followed by quantitative analysis. Results: Analyses of the connective tissue revealed a significantly higher number of CD3-expressing T cells and CD11c-expressing dendritic cells in the connective tissue of patients with OFG-S compared to patients with OFG+CD. Mast cells displayed a high level of activation, although no significant difference was detected when comparing the two groups. Conclusions: The results show a different composition of the inflammatory infiltrate in patients with OFG-S compared to patients with OFG+CD. The present observations support that partly divergent immune mechanisms are involved in these two different subcategories of OFG.

** Key words:**Granulomas, autoimmunity, T cells, B cells, dendritic cells, children, adults.

## Introduction

Orofacial granulomatosis (OFG) is a rare inflammatory disorder characterised by oral lesions such as lip/facial swelling, angular cheilitis, cobblestone phenomenon, tag formation, full thickness gingivitis and ulceration (1). OFG has been associated with systemic diseases such as Crohn’s disease (CD) (2) and with food hypersensitivity reactions (3-5).

Current knowledge implicates a complex involvement of different types of immune cells in patients with OFG and CD, and T cells have been attributed a major role in the pathogenesis of these two disorders (6,7). Dendritic cells (DCs) influence the fate decisions of naive CD4-expressing T cells towards Th1, Th2 or Th17 subsets at the early stages of activation (8,9). Two major subsets of DCs reside in the oral mucosa, CD1a-positive Langerhans cells (LCs) and CD11c-positive lamina propria resident DCs (10). However, the influence of DCs in the pathogenesis of OFG has not been fully elucidated.

In patients with OFG with or without concurrent CD, the presence of non-caseating granulomas in tissue specimens from affected areas is considered to be a hallmark of histopathologically proven diseases (1). In both OFG and CD, granulomas are characterised by the presence of multinucleated giant cells of macrophage origin (7,11) that express CD68 molecules. The underlying mechanisms of granuloma formation in the two conditions are still unknown.

It has been suggested that IgE-expressing B cells in the submucosa of OFG patients play an important role in the pathogenesis and explain previous findings suggesting a link between food allergy and OFG (12). Mast cells are important players in IgE-mediated reactions, including food-elicited conditions (13). So far the role of mast cells in OFG has not been thoroughly investigated.

OFG may be considered a separate disease entity when appearing exclusively in the oral region. When it exists in conjunction with a systemic disease, it may represent a distinct subcategory. Immune mechanisms and the reflecting immunohistochemistry may partly differ between these subcategories. Therefore, the aim of this investigation was to characterise and compare the immune cell infiltration in two groups of patients with OFG, one without systemic diseases (OFG-S) and the other one with coexisting CD (OFG+CD).

## Patient and Methods

-Patients and samples

A total of 22 OFG patients (OFG-S [n=11] and OFG+CD [n=11]) with a median age of 16 years (range 8–51 years) were included ( Table 1). The clinical inclusion criteria for OFG in both groups were lip and/or facial swelling together with tag formation, cobblestone phenomenon and/or angular cheilitis. The diagnosis of paediatric and adult CD was confirmed according to established criteria (14,15). In case of gastrointestinal symptoms, the patients were subjected to gastrointestinal endoscopy with special focus on CD. No sarcoidosis or proven food-induced OFG were found in any of the patients. Two patients in the OFG+CD group received mesalazine (PentasaTM, Ferring, Saint-Prex, Switzerland) and all the other patients were free from medication. Extended-release mesalamine granules are known to have a local effect in the gut mucosa (16). The histopathological inclusion criterion was the presence of non-caseating granulomas in at least one biopsy at any location of the oral mucosa ( Table 1). In this retrospective study, biopsies were taken from the most prominent OFG manifestation site: gingivae (n=8), bucca (n=7), sulcus (n=7) and the lip (n=5).

**Table 1:**
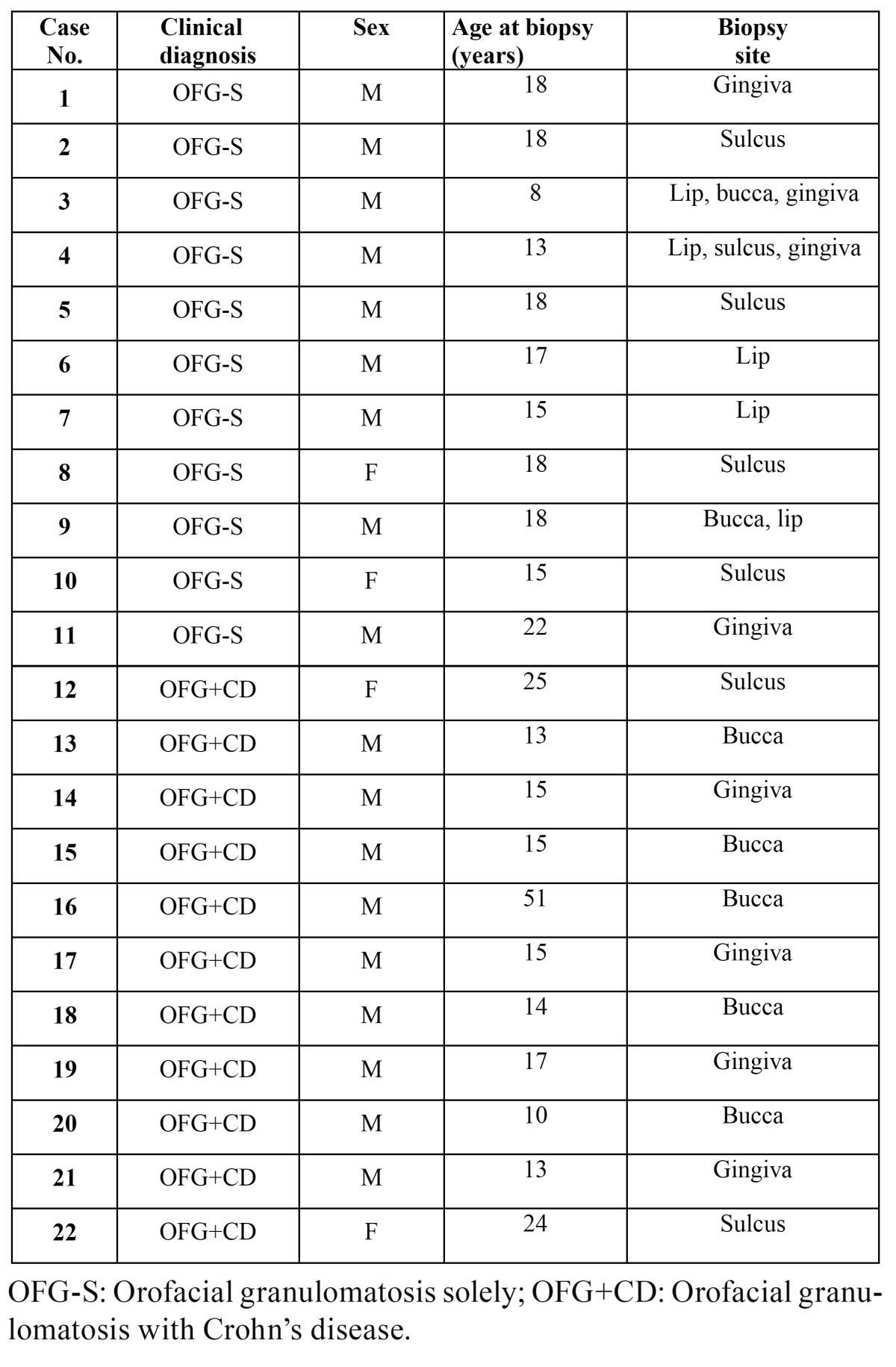
Patient characteristics.

-Immunohistochemistry

Antibodies: Antihuman CD1a (clone 010, isotype IgG1, kappa), CD20 (clone L26, isotype IgG2a, kappa), mouse-monoclonal primary antibody anti-CD68, (clone KP1, isotype IgG1, kappa) and mast cell tryptase (MCT; clone AA1, isotype IgG1, kappa) were purchased at Dako Sweden AB. Monoclonal mouse antibodies antihuman CD3 (clone F7.2.38, isotype IgG1, kappa) and monoclonal rabbit CD11c (clone EP1347Y, isotypeIgG) was obtained from AbCam, Cambridge, United Kingdom. Monoclonal antibody CD4 (clone 4B12, NCL-CD4-368) and monoclonal antibody CD8 (clone 4B11, NCL-CD8-4B11) were purchased at Novocastra, Leica Microsystems AB, Sweden.

Immunostaining: Paraffin-embedded tissue specimens were cut at 4 µm thick sections and mounted on DakoIHC microscope slides, deparaffinised and then re-dehydrated. Endogenous peroxidase was blocked with 3% hydrogen peroxide for 5 min, washed in buffer (0.1% phosphate buffered saline (PBS) + Triton-X-100 0.05%) for 5 min and followed by block non-specific proteins with 10% normal serum in 0.5% PBS for 20 min. For CD4 antigen retrieval in Tris-EDTA pH 9.0 was done in microwave oven for 10 min followed by blocking with Background Sniper (Biocare, Concord, CA, USA, BS966M) for 15 min. Staining with primary antibody against CD1a, CD3 (dilution 1:100), CD4 (dilution 1:200), CD8 (dilution 1:100), CD11c (dilution 1:65), CD20 (dilution 1:200), CD68 (dilution 1:1000) and mast cell tryptase (MCT) (dilution 1:1500) in 1% biotinylated secondary antibody (BSA) was performed for 60 min and wash in buffer for 2×5 min, followed by an incubation with BSA (Vector, Burlingame, CA 94110, USA) for 30 min and wash in buffer for 2×5 min. For CD4 Mach3MouseProbe (Biocare, M3M532L) was used as secondary antibody. Incubation with avidinbiotin complex (Vector ABC: 2.5 ml PBS + 50 μl A + 50 μl B) for 30 min was performed followed by buffer wash for 2×5 min. DAB (DAB: One set of Sigma tablets to 5 ml of distilled H2O) was added to slides for 2 min followed by washing in distilled H2O several times. For CD4 a tertiary step was performed using Mach3MouseAP-polymer (Biocare, M3M532L) for 30 min and Vulcan Fast Red substrate (Biocare, FR805S) for 2 min with levamisol (Vector, SP-5000). Background staining in Mayer’s hematoxylin was carried out for 40 seconds followed by blueing in tap water for 4 min. Finally, the sections were dehydrated and mounted with Pertex (Histolab Products AB, Spånga, Sweden). All steps were done at room temperature.

Quantitative analysis was performed on a minimum of two areas per tissue section with an average of four areas per patient, apart from one patient where the biopsy was too small to count two different sections. Digitalised images from one to eight fields, dependent on biopsy size, at a magnification of ×80 were obtained using a light microscope (LeitzWetzler, Leica Microsystems, Wetzlar, Germany) equipped with a UC30 Olympus camera (Olympus Microsystems, Norcross, GA 30071, USA). The sections were then analysed using the computer software BioPixiQ 2.0 (BioPix, Gothenburg, Sweden), where the percentage of stained tissue area was calculated as previously described (17). The areas of positively stained cells within the connective tissue containing granulomas were registered. These areas of positive cells were then expressed as a percentage of the connective tissue area. In addition to the quantitative analyses of mast cells using this method, analyses of mast cell degranulation were performed with a semi quantitative scale where the amount of released tryptase-positive granules was assessed: 0: no released granules, 1: low number of released granules (1-3), 2: moderate number of released granules (4-9) and 3: high number (10 or above) of released granules. Mast cell-specific proteases include tryptases, chymases and carboxypeptidase A, but as tryptases are the most abundant of mediators found in granules from mast cells, we used tryptase as a marker for mast cells. The number of de granulated cells was expressed as a percentage of all tryptase-positive cells. For statistical analysis, data in scale steps 0 + 1 and 2 + 3 were merged.

-Statistical analyses

Analyses of differences between groups were calculated by the Mann-Whitney U-test, utilising the statistical software GraphPad Prism Version 6.0b (GraphPad Software, Inc., San Diego, CA, USA). A *p*-value <0.05 was considered as a significant difference.

This study was approved by the Ethics Committee at The Sahlgrenska Academy, University of Gothenburg and informed consent was obtained from all patients, and when needed, also from their parents.

## Results

**Figure 1:**
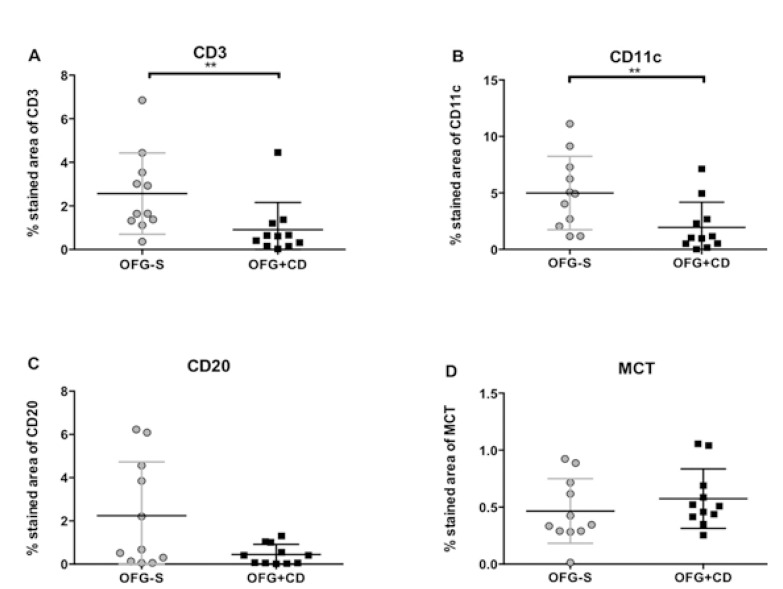
Positively stained area (%) of connective tissue in patients with orofacial granulomatosis solely (OFG-S; circles) and orofacial granulomatosis with Crohn´s Disease (OFG+CD; squares) for A) CD3-expressing T cells, B) CD11c-expressing cells, C) CD20-expressing cells and D) tryptase (MCT)-expressing mast cells. Median values and interquartile ranges are indicated. ** *P* < 0.01.

Immunohistochemical staining of biopsies from the 11 patients with OFG-S and the 11 patients with OFG+CD was performed using markers for CD1a, CD3, CD4, CD8, CD11c, CD20, CD68 and MCT. The results of CD3, CD11c, CD20 and MCT positively stained cells in the connective tissues are displayed in figure 1.

-T-cells 

CD3-positiveT cells were found in the connective tissue from both OFG-S and OFG+CD patients in a similar distribution pattern and with T cells evenly distributed in the connective tissue (Fig. 2A-B) as well as clustering around the granulomas (Fig. 2A).There was a significantly higher number of CD3-expressing T cells, as reflected by positively stained areas, in the connective tissues in patients with OFG-S compared to patients with OFG+CD (Fig. 1A; *p* = 0.005).

**Figure 2:**
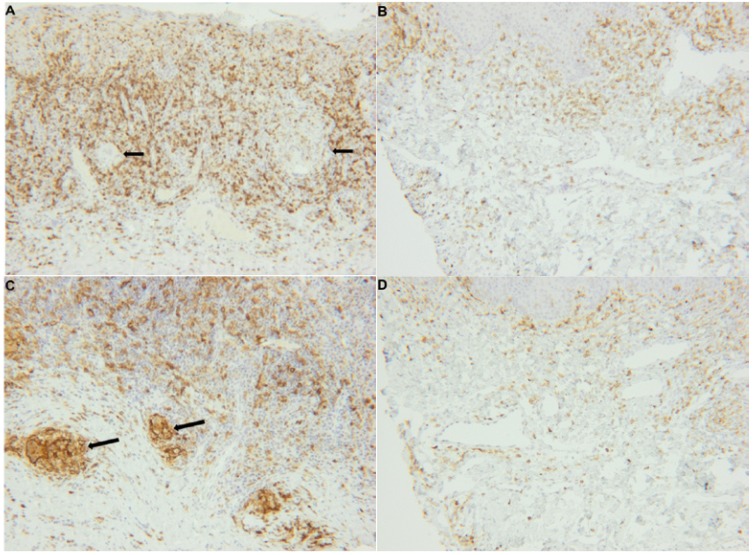
Immunostaining panel of connective tissue in patients with OFG-S and OFG+CD. CD3 expressing cells in A) OFG-S and B) OFG+CD, CD11c expressing cells in C) OFG-S and D) OFG+CD,

CD4-expressing cells with lymphocyte morphology could be observed both within the granulomas and evenly spread out in the connective tissue. In some tissue sections CD4-positive cells could also be detected in the epithelium. There was no significant difference in the presence of CD4-positive cells between the OFG-S and OFG+CD groups. Neither was there any difference in the presence of CD8-positive cells between the two groups and similar tissue distribution as for CD4-positive cells was observed.

-Dendritic cells and macrophages

In the connective tissue the CD1a-positive Langerhans cells (LCs) were evenly distributed, while in the epithelium LCs were observed in clusters and had a more prominent dendritic appearance. There was no significant difference in the number of LCs in patients with OFG-S compared to OFG+CD patients.

CD11c molecules were mostly expressed by non-dendritic multinucleated giant cells present in the granulomas (Fig. 2C). This marker could also be observed in other parts of the connective tissue (Fig. 2D), but then expressed by cells with a dendritic morphology. CD11c-positive cells were significantly more abundant in the OFG-S group compared to the OFG+CD group (Fig. 1B; *p*=0.007).

CD68-expressing macrophages were seen distributed in the connective tissue including granulomas. Regarding

the number of CD68-expressingcells, there was no significant difference between the two groups.

-B-cells 

The distribution of CD20 molecule-expressing B cells varied between biopsies; in some cases a homogeneous distribution was noted (Fig. 2*E-F), while in other cases an accumulation surrounding granulomas was observed. No significant difference could be seen regarding numbers, although there was a trend towards more B cells in the OFG-S group compared to the OFG+CD group (*p*=0.087; Fig. 1C). In general, the CD20-expressing cells showed lymphocyte morphology, although to some extent with a more irregular appearance than to be expected by tissue-resident lymphocytes (Fig. 3A-B).

**Figure 2*:**
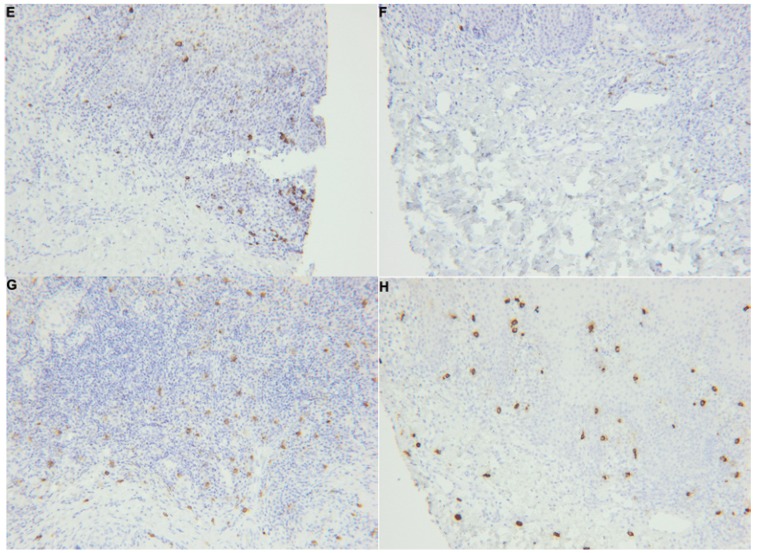
CD20 expressing cells in E) OFG-S and F) OFG+CD. MCT-expressing cells in G) OFG-S and H) OFG+CD.

**Figure 3:**
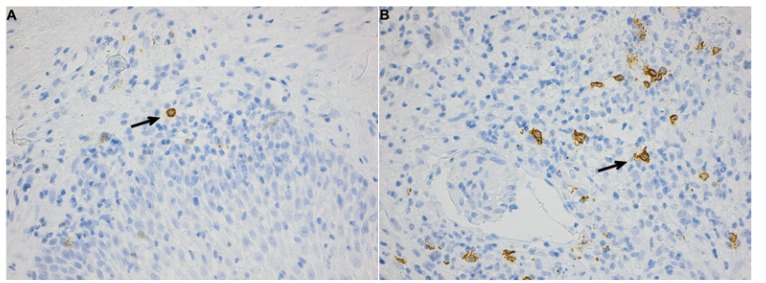
CD20- expressing B cells (arrows) with A) lymphocytic morphology and B) with a more irregular morphology. Magnification x400.

-Mast cells

**Figure 4:**
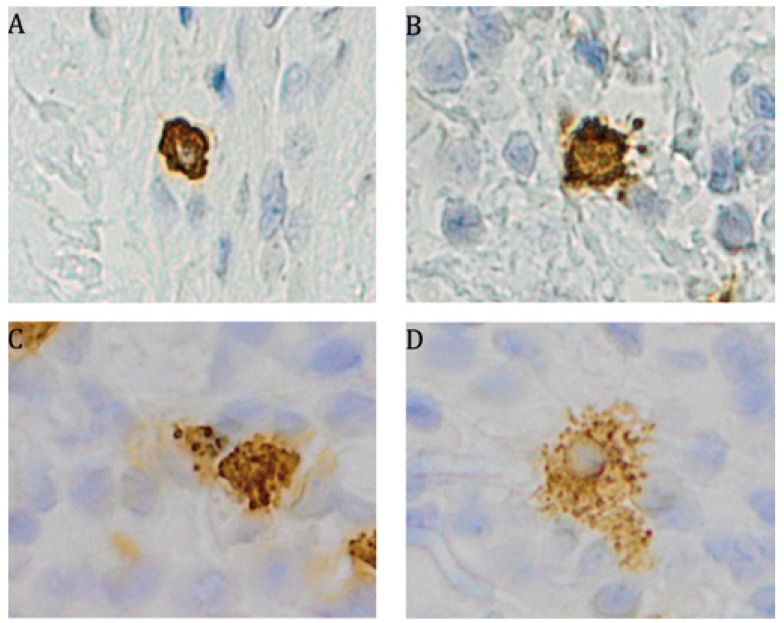
Immunostainings of tryptase positive granule-releasing mast cells.Mast cell with A) no degranulation (0 granules), B) low number of granules (1-3), C) moderate number of granules (4-9) and D) high number of relase of granules (10 or above). Magnification ×400.

Mast cells were found in both the OFG-S and the OFG+CD groups and notably showed a high degree of mast cell tryptase release in both groups (Fig. 2* )G-H . No significant difference could be detected when comparing the total frequency of granule-releasing mast cells in the OFG-S group with the OFG+CD group (Fig. 1D). When looking at levels of granule release, the no granule group (Fig. 4A) and the low granule group (Fig. 4B) were merged, with no significant difference between patients with OFG-S and OFG+CD ( Table 2). Neither was any difference observed between OFG-S and OFG+CD (Table 2) when merging the moderate (Fig. 4C) and the high number (Fig. 4D) granule groups.

**Table 2:**
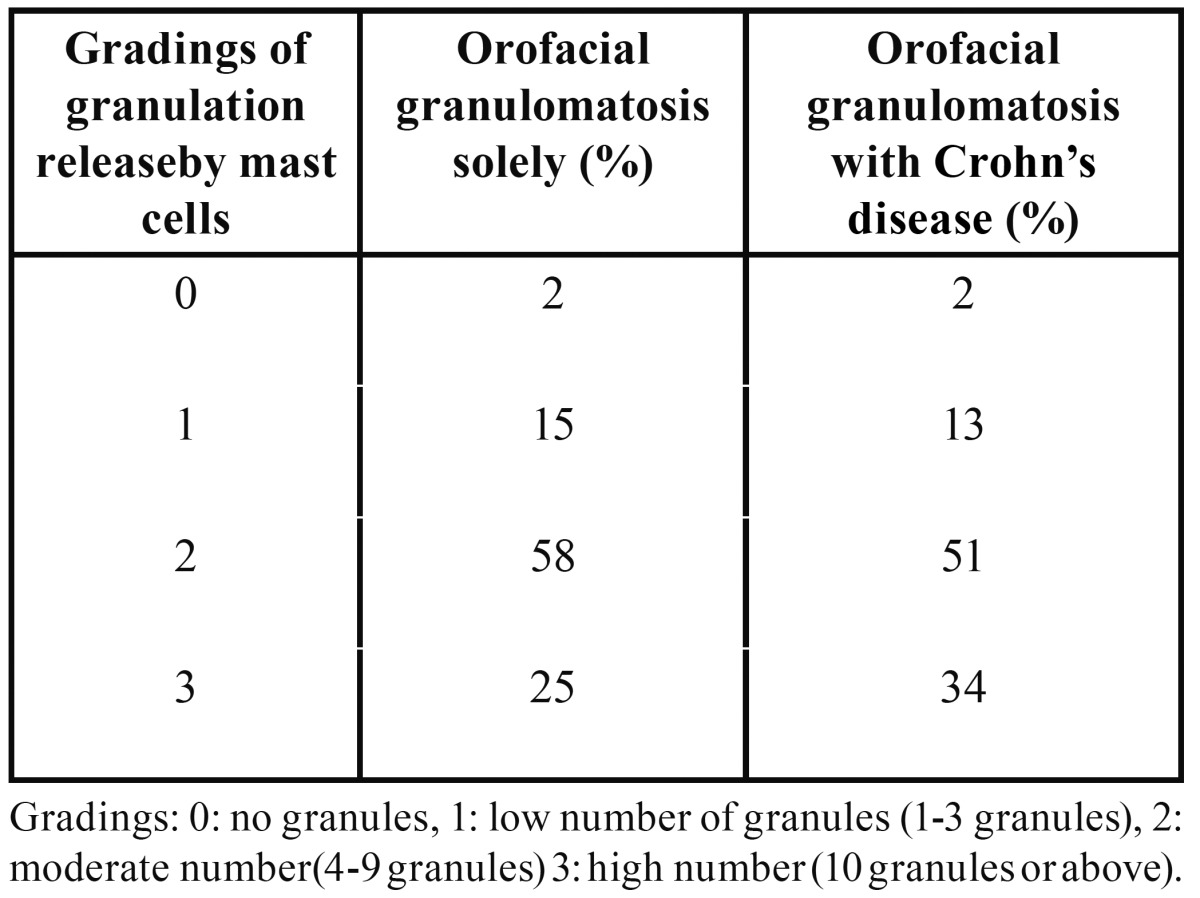
Semi quantification of granulation release by mast cells in % of all mast cells.

-Epitheloid cell granulomas

According to the inclusion criteria, all biopsies from the 22 OFG patients showed granuloma formation with multinucleated giant cells and lymphocytes observed by routine hematoxylin-eosine histology. Immunostaining showed that the multinucleated giant cells expressed CD68, the marker for macrophages. In both groups, approximately 10% of the area in the connective tissue represented granulomas.

## Discussion

In this study we compared various cell populations in oral lesions of patients with OFG solely (OFG-S) and OFG with concomitant CD (OFG+CD). T cells were present in higher numbers in lesions from patients with OFG-S compared to patients with OFG+CD.Furthermore, lesions from patients with OFG-S had higher numbers of CD11c-expressing dendritic cells than those in patients with OFG+CD. These observations support the view that these two subcategories of OFG have immunopathogenic differences.

Both OFG (1) and CD (18) belong to the group of granulomatous diseases, characterised by the presence of non-caseating granulomas consisting of multinucleated giant cells expressing CD68 molecules (7,11). Although granuloma is not a prerequisite for the diagnosis of OFG, it strengthens the present study in that only OFG patients with granuloma in their oral biopsies were included. This provides greater diagnostic accuracy and probably also more uniform and representative observations of the OFG inflammation.

We show that CD3-positive T cells are present in higher amounts in tissue specimens from patients with OFG-S than those in patients with OFG+CD. Regarding OFG-S, this is well in line with the results presented by Freysdottir and coworkers, who observed T cells both in and outside granulomas in biopsies from patients with OFG (7). Despite a significant difference in the percentage of CD3-positive Tcells between the two patient groups, we were not able to find any differences in the percentages of the subsets of CD4-positive or CD8-positivecells.This may be due to interference from CD4 molecules expressed by DCs, granulocytes and macrophages, making a valid comparison difficult (19,20).

Two major subsets of DCs reside in oral mucosa, CD1a-positive Langerhans cells (LCs) and CD11c-positive lamina propria resident DCs (10). Both these antigen-presenting cells may be of importance in disease pathology. In this study the presence of CD1a-expressing LCs in connective tissue from OFG-S and OFG+CD patients did not differ. However, our impression is that the number of CD1a-expressing cells is increased in comparison to healthy oral connective tissue. This is in line with our previous studies showing higher numbers of CD1a-expressing LCs in connective tissue of other oral inflammatory disorders (lichen planus and graft-versus-host disease) compared to healthy oral mucosa (21,22).

Granulomas are considered to be sites of persistent antigen localisation (23), where the immune system tries to eliminate deleterious antigens (24). In a recent experimental study of mycobacterial granulomas, DCs were found to traffic in and out of granulomas (25), indicating a central role for this cell subset in disease pathogenesis. We observed that CD11c-positive DCs were present in the lamina propria of the OFG-S patients in higher numbers than in patients with OFG+CD, which may indicate that DCs participate differently in the pathogenesis of OFG-S compared to OFG+CD. The influx of DCs in OFG-S patients may be a reflection of an oral reaction pattern involving an exposure to exogenous antigens such as food constituents or bacterial components. Specific food constituents have been attributed a triggering role in OFG (3,26,27), and a continuous exposure to food antigens may in a sensitised individual lead to the recruitment of DCs to oral tissues.

B cells have previously been found in OFG (12) and here we show that CD20-expressing B cells are found in the connective tissue with a heterogeneous distribution. Thus, tissue lesions from patients with OFG-S seem to recruit more antigen-presenting cells, more T cells and a trend towards more B-cells than disease affected tissues from patients with OFG+CD.

Importantly, a Th2-skewed T cell response regulates B cell class switching to IgE and mast cell activation (28). The occurrence of B cell class switching to IgE has been reported in OFG (12). Our findings show a great number of mast cells with released granules that may be the result of IgE-mediated mast cell activation. In both the OFG-S and OFG+CD groups, high mast cell activity was registered, although no significant difference between the two groups was recorded. Increased frequencies of mast cells have also been reported in gut mucosa from patients with CD, and these cells have been implicated in the pathogenesis of this disease (13). Food antigens as a triggering factor may be considered, but other exogenous stimuli may also be of importance. In a recent paper, Nakamura *et al*. provided evidence for Staphylococcus delta-toxin causing mast cell degranulation in patients with atopic dermatitis (29). In OFG a similar microbial-induced reaction might be of relevance, especially for the perioral and lip lesions. Thus, the high mast cell activity in both OFG-S and OFG-CD may be an important finding with clinical implications.

OFG-S and OFG-CD patients investigated in this study display a skewed gender distribution with a predominance of males. This is in accordance with our recently published Swedish cohort study, where the male:female ratio was approximately 3:1 (30). Furthermore, in a study from Sweden on pediatric patients with CD, male patients dominated (31). In contrast, studies from other geographic areas report a more homogenous gender distribution of OFG with and without CD (12,32,33) with male:female ratios of approximately 1:1.

OFG-S and OFG+CD may represent two subcategories that could differ in various aspects, such as immune mechanisms, clinical characteristics and genetics. As an example, Savage and co-workers found that serum IgA antibodies to S. cerevisiae were raised significantly in the OFG+CD but not in the OFG-S group (34). Regarding clinical characteristics, we have recently observed that OFG-S patients perceived their overall discomfort, aesthetic problems and social discomfort as more severe than OFG+CD patients. Furthermore, the two groups seemed to differ regarding genetic characteristics, that is, NOD2 gene variation pattern (30). In conclusion, the present study reinforces the concept of OFG as a heterogeneous clinical entity.

## References

[1] Wiesenfeld D, Ferguson MM, Mitchell DN, MacDonald DG, Scully C, Cochran K (1985). Oro-facial granulomatosis--a clinical and pathological analysis. Q J Med.

[2] Plauth M, Jenss H, Meyle J (1991). Oral manifestations of Crohn's disease. An analysis of 79 cases. J Clin Gastroenterol.

[3] Saalman R, Mattsson U, Jontell M (2009). Orofacial granulomatosis in childhood-a clinical entity that may indicate Crohn's disease as well as food allergy. Acta paediatrica.

[4] Fitzpatrick L, Healy CM, McCartan BE, Flint SR, McCreary CE, Rogers S (2011). Patch testing for food-associated allergies in orofacial granulomatosis. J Oral Pathol Med.

[5] Campbell H, Escudier MP, Brostoff J, Patel P, Milligan P, Challacombe SJ (2013). Dietary intervention for oral allergy syndrome as a treatment in orofacial granulomatosis: a new approach?. J Oral Pathol Med.

[6] Facchetti F, Signorini S, Majorana A, Manganoni MA, Sapelli P, Imberti L (2000). Non-specific influx of T-cell receptor alpha/beta and gamma/delta lymphocytes in mucosal biopsies from a patient with orofacial granulomatosis. J Oral Pathol Med.

[7] Freysdottir J, Zhang S, Tilakaratne WM, Fortune F (2007). Oral biopsies from patients with orofacial granulomatosis with histology resembling Crohn's disease have a prominent Th1 environment. Inflammatory bowel diseases.

[8] Stockinger B, Veldhoen M, Martin B (2007). Th17 T cells: linking innate and adaptive immunity. Seminars in immunology.

[9] Yamane H, Paul WE (2013). Early signaling events that underlie fate decisions of naive CD4(+) T cells toward distinct T-helper cell subsets. Immunological reviews.

[10] Hovav AH (2014). Dendritic cells of the oral mucosa. Mucosal immunology.

[11] Miura M, Hiwatashi N, Yamashita K, Kimura M (1989). Immunohistologic characterization of epitheloid cells in Crohn's granulomas. Journal of clinical & laboratory immunology.

[12] Patel P, Barone F, Nunes C, Boursier L, Odell E, Escudier M (2010). Subepithelial dendritic B cells in orofacial granulomatosis. Inflammatory bowel diseases.

[13] Hagel AF, deRossi T, Zopf Y, Konturek P, Dauth W, Kressel J (2013). Mast cell tryptase levels in gut mucosa in patients with gastrointestinal symptoms caused by food allergy. International archives of allergy and immunology.

[14] Dignass A, Van Assche G, Lindsay JO, Lemann M, Soderholm J, Colombel JF (2010). The second European evidence-based Consensus on the diagnosis and management of Crohn's disease: Current management. Journal of Crohn's & colitis.

[15] Levine A, Griffiths A, Markowitz J, Wilson DC, Turner D, Russell RK (2011). Pediatric modification of the Montreal classification for inflammatory bowel disease: the Paris classification. Inflammatory bowel diseases.

[16] Criscuoli V, Modesto I, Orlando A, Cottone M (2013). Mesalazine for the treatment of inflammatory bowel disease. Expert opinion on pharmacotherapy.

[17] Raghavan S, Ostberg AK, Flach CF, Ekman A, Blomquist M, Czerkinsky C (2010). Sublingual immunization protects against Helicobacter pylori infection and induces T and B cell responses in the stomach. Infection and immunity.

[18] Williams WJ (1964). Histology of Crohn's Syndrome. Gut.

[19] Biswas P, Mantelli B, Sica A, Malnati M, Panzeri C, Saccani A (2003). Expression of CD4 on human peripheral blood neutrophils. Blood.

[20] Gibbings D, Befus AD (2009). CD4 and CD8: an inside-out coreceptor model for innate immune cells. Journal of leukocyte biology.

[21] Gustafson J, Eklund C, Wallstrom M, Zellin G, Magnusson B, Hasseus B (2007). Langerin-expressing and CD83-expressing cells in oral lichen planus lesions. Acta odontologica Scandinavica.

[22] Hasseus B, Jontell M, Brune M, Johansson P, Dahlgren UI (2001). Langerhans cells and T cells in oral graft versus host disease and oral lichen planus. Scandinavian journal of immunology.

[23] Chambers TJ, Morson BC (1979). The granuloma in Crohn's disease. Gut.

[24] Ito T, Connett JM, Kunkel SL, Matsukawa A (2013). The linkage of innate and adaptive immune response during granulomatous development. Frontiers in immunology.

[25] Schreiber HA, Harding JS, Hunt O, Altamirano CJ, Hulseberg PD, Stewart D (2011). Inflammatory dendritic cells migrate in and out of transplanted chronic mycobacterial granulomas in mice. The Journal of clinical investigation.

[26] Campbell HE, Escudier MP, Patel P, Challacombe SJ, Sanderson JD, Lomer MC (2011). Review article: cinnamon- and benzoate-free diet as a primary treatment for orofacial granulomatosis. Alimentary pharmacology & therapeutics.

[27] Patel P, Brostoff J, Campbell H, Goel RM, Taylor K, Ray S (2013). Clinical evidence for allergy in orofacial granulomatosis and inflammatory bowel disease. Clinical and translational allergy.

[28] Paul WE, Zhu J (2010). How are T(H)2-type immune responses initiated and amplified?. Nature reviews Immunology.

[29] Nakamura Y, Oscherwitz J, Cease KB, Chan SM, Munoz-Planillo R, Hasegawa M (2013). Staphylococcus delta-toxin induces allergic skin disease by activating mast cells. Nature.

[30] Gale G, Ostman S, Rekabdar E, Torinsson Naluai A, Hogkil K, Hasseus B Characterisation of a Swedish cohort with orofacial granulomatosis with or without Crohn’s disease. Health Econ.

[31] Malmborg P, Grahnquist L, Lindholm J, Montgomery S, Hildebrand H (2013). Increasing incidence of paediatric inflammatory bowel disease in northern Stockholm County, 2002-2007. Journal of pediatric gastroenterology and nutrition.

[32] McCartan BE, Healy CM, McCreary CE, Flint SR, Rogers S, Toner ME (2011). Characteristics of patients with orofacial granulomatosis. Oral diseases.

[33] Sanderson J, Nunes C, Escudier M, Barnard K, Shirlaw P, Odell E (2005). Oro-facial granulomatosis: Crohn's disease or a new inflammatory bowel disease?. Inflammatory bowel diseases.

[34] Savage NW, Barnard K, Shirlaw PJ, Rahman D, Mistry M, Escudier MP (2004). Serum and salivary IgA antibody responses to Saccharomyces cerevisiae, Candida albicans and Streptococcus mutans in orofacial granulomatosis and Crohn's disease. Clinical and experimental immunology.

